# Short-term difference only in reported outcomes (PROMs) after anterior or posterior approach to total hip arthroplasty: a 4-year prospective multi-centre observational study

**DOI:** 10.1186/s13018-023-03603-0

**Published:** 2023-02-17

**Authors:** D-Yin Lin, Anthony J. Samson, Matthew G. Cehic, Brigid Brown, Billingsley Kaambwa, Christopher Wilson, Hidde M. Kroon, Ruurd L. Jaarsma

**Affiliations:** 1 Department of Anesthesiology, Flinders Medical Centre, Flinders Drive, Bedford Park, Adelaide, SA 5042 Australia; 2 College of Medicine and Public Health, Flinders University, Adelaide, SA Australia; 3 Department of Orthopaedic and Trauma Surgery, Flinders Medical Centre, Adelaide, SA Australia; 4 Health Economics, College of Medicine and Public Health, Flinders University, Adelaide, SA Australia; 5 Department of Surgery, Royal Adelaide Hospital, Adelaide, SA Australia; 6 Discipline of Surgery, Adelaide Medical School, Faculty of Health and Medical Sciences, University of Adelaide, Adelaide, SA Australia

## Abstract

**Background:**

The direct anterior approach (DAA) in total hip arthroplasty (THA) may demonstrate better functional recovery compared to the posterior approach (PA).

**Methods:**

In this prospective multi-centre study, patient-related outcome measures (PROMs) and length of stay (LOS) were compared between DAA and PA THA patients. The Oxford Hip Score (OHS), EQ-5D-5L, pain and satisfaction scores were collected at four perioperative stages.

**Results:**

337 DAA and 187 PA THAs were included. The OHS PROM was significantly better in the DAA group at 6 weeks post-operatively (OHS: 33 vs. 30, *p* = 0.02, EQ-5D-5L: 80 vs. 75, *p* = 0.03), but there were no differences at 6 months and at 1 year. EQ-5D-5L scores were similar between both groups at all time points. LOS as inpatient was significantly different, in favour of DAA [median 2 days (IQR 2–3) vs. PA 3 (IQR 2–4), *p* ≤ 0.0001].

**Conclusions:**

Patients undergoing DAA THA have shorter LOS and report better short-term Oxford Hip Score PROMs at 6 weeks, but DAA did not convey long-term benefits over PA THA.

**Supplementary Information:**

The online version contains supplementary material available at 10.1186/s13018-023-03603-0.

## Introduction

The direct anterior approach (DAA) to facilitate total hip arthroplasty (THA) was first described by Carl Heuter in 1881 [1]. This is a minimally invasive technique that utilises the tissue plane between the tensor fascia lata and rectus femoris [2]. It was later modified into the Smith–Petersen method [3], with increasing uptake in recent years. Some evidence suggests that the DAA results in improved early functional recovery and lower post-operative pain scores, when compared to the more traditional posterior approach (PA), but these results have not been consistently supported [4–6]. Furthermore, there is a paucity of evidence for long-term outcomes supporting DAA as a standard approach for THA [7–9].

In this study, we aimed to determine if DAA THA resulted in improved patient-reported quality of recovery, shorter LOS and lower long-term pain outcomes compared to PA THA.

## Patients and methods

This multi-centre prospective study was conducted across two tertiary teaching hospitals in Adelaide, Australia. Orthopaedic surgeons operate routinely at both hospitals, performing approximately 500 arthroplasty procedures per year. Due to SARS Covid-19 related restrictions on elective operations, this number was reduced in 2020 to approximately 300. Each orthopaedic surgeon has a preference for performing DAA or PA, and hence, both approaches were performed by different surgeons. Allocation of patient to surgeon is determined in a multidisciplinary discussion, with a group of admitting orthopaedic surgeons present representing proficiency in both approaches. Each individual patient would then be allocated to either DAA or PA based on patient characteristics, pathology and roster availability of supervising surgeon. The data include DAA and PA training curves for both some attending surgeons as well as senior trainee surgeons. One hundred per cent of cases were supervised by a senior consultant orthopaedic surgeon experienced in the applied approach, and in 70% of cases, a training consultant surgeon, fellow or senior trainee were the supervised primary surgeon. No operations were performed by junior medical staff.

All consecutive adult patients at both hospitals undergoing elective THA were prospectively enrolled over a 3-year period from 8th January 2018 to 1st of October 2020, with a year follow-up until 2nd October 2021. Indication for elective THA was for the vast majority osteoarthritis, zero trauma patients were included. The local Human Research Ethics Committee granted multi-centre approval (SALHN/329.17). Informed consent was obtained from all participants. This trial was retrospectively registered (Netherlands Trial Registry: NL9803).

Data for this study were recorded by a dedicated research assistant, using scripted questionnaires either via telephone or via a posted written survey. The same script was used at four different time points: pre-operatively, and post-operatively at 6 weeks, 6 months and 1 year. At all four times, two validated patient-reported outcome measures (PROMs) were used: the Oxford Hip Score (OHS) [10, 11] and the EQ-5D-5L Health Questionnaire, including pain/discomfort and anxiety/depression scores [12]. These have previously been used in similar studies for THA outcomes [13]. Also recorded were LOS, opioid use at all four time points as well as a 5-point Likert scale for patient satisfaction post-operatively. Data were entered into a password secured database stored on the hospital computer network.

### Statistical analysis

The analysis was performed using SPSS version 27 (IBM Corp., Armonk, NY, USA) and GraphPad Prism version 8 (GraphPad Software, La Jolla, Calif, USA). Continuous variables were tested for parametricity (Shapiro–Wilk test). Nonparametric variables are described as median with interquartile range (IQR). Univariate analysis was carried out using the chi-squared test for categorical variables, and the Mann–Whitney *U*-test for nonparametric continuous variables. A *p* value of < 0.05 was considered statistically significant.

## Results

A total of 557 consecutive patients were approached to participate, of whom 527 (95%) provided informed consent. Eight patients were excluded due to use of an approach other than DAA or PA (namely lateral), the remaining 519 were included in this analysis: 337 underwent a DAA, and 182 a PA.

There were more female patients in the DAA group than in the PA group [204 (60.5%) vs. 93 (51.1%), respectively, *p* = 0.038], but no difference in median age [70 year (IQR 63–76) vs. 71 (60–79), respectively, *p* = 0.18]. The median body mass index (BMI) was lower in the DAA group than in PA [29.6 kg/m^2^ (IQR 26.0–34.0) vs. 31.3 (27.0–35.9), respectively, *p* = 0.005] (Table 1).

**Table 1:** Baseline characteristics for anterior and posterior hip arthroplasty patients

	Anterior (*n* = 337)	Posterior (*n* = 182)	*p* value
Age in years, median (IQR)	70 (63–76)	71 (60–79)	0.18^a^
Gender, *n* (%)			
Male	133 (39.5)	89 (48.9)	**0.038**^b^
Female	204 (60.5)	93 (51.1)	
Weight in kg, median (IQR)	82.5 (71.3–93.8)^c^	83.2 (70.0–98.0)^d^	0.37^a^
BMI in kg/m^2^, median (IQR)	29.6 (26.0–34.0)^e^	31.3 (27.0–35.9)^f^	**0.005**^a^
Operative side, *n* (%)			
Left	150 (44.5)	82 (45.0)	0.91^b^
Right	187 (55.4)	100 (55.0)	

Bold denotes statistical significance

*IQR* interquartile range

^a^Mann–Whitney U test

^b^Chi-squared test

^c^*n* = 190

^d^*n* = 129

^e^*n* = 299

^f^*n* = 160

Length of stay as an inpatient was significantly different between the two groups, *p* ≤ 0.0001. Patients who underwent a DAA had a shorter LOS of 2 days, IQR 2–3. PA patients had a median of 3 days IQR 2–4.

Use of pre-operative opioid-based medication was similar between both groups [DAA 108 patients (32%) vs. PA 55 (30%), *p* = 0.67], but the DAA group had more pre-operative pain/discomfort than the PA cohort [254 (76%) patients had severe or extreme pain vs. 122 (67%), respectively, *p* = 0.02] (Table 2). No patient in either group used post-operative opioid-based medication at 6 weeks, 6 months and 1 year. The Oxford Hip Scores were similar between both groups pre-operatively and showed a median post-operative improvement of 21 points at 6 weeks in the DAA group (33 points, IQR 25–38), which was significantly higher than the 18-point improvement in the PA group (30 points, IQR 23–36, *p* = 0.02). At 6 months and 1 year, there was no difference in the Oxford Hip Scores between the two groups: the DAA group had a median score of 41 [IQR 33–45) at 6 months, which was 37 (IQR 30–44) in the PA group (*p* = 0.10) and at 1 year, this was 44 points for both groups (*p* = 0.56] (Table 2).

**Table 2:** Pre- and post-operative opiate use, and patient-reported outcome measures (PROMs) for anterior and posterior hip arthroplasty

	Anterior (*n* = 337)	Posterior (*n* = 182)	*p* value
Opiate use, *n* (%)^a^			
Pre-operative	108 (32)	55 (30)	0.67^a^
6 weeks post-operative	0 (0)^c^	0 (0)^f^	–
6 months post-operative	0 (0)^d^	0 (0)^g^	–
1 year post-operative	0 (0)^e^	0 (0)^h^	–
Oxford Hip Score total, median (IQR)^b^			
Pre-operative	12 (8–20)	12 (7–18)	0.41^b^
6 weeks post-operative	33 (25–38)^c^	30 (23–36)^f^	**0.02**^b^
6 months post-operative	41 (33–45)^d^	37 (30–44)^g^	0.10^b^
1 year post-operative	44 (40–47)^e^	44 (33–48)^h^	0.56^b^
EQ-5L-5D Health Questionnaire Utility Scores, median (IQR)^b^			
Pre-operative	− 0.030 (− 0.676 to 0.911)	− 0.024 (− 0.176 to 0.367)	0.47^b^
6 weeks post-operative	0.672 (0.521–0.805)^c^	0.672 (0.502–0.805)^f^	**0.69**^b^
6 months post-operative	0.860 (0.661–1.000)^d^	0.884 (0.661–1.000)^g^	0.70^b^
1 year post-operative	1.000 (0.860–1.000)^e^	1.000 (0.733–1.000)^h^	0.07^b^

Bold denotes statistical significance

*IQR* interquartile range

^a^Chi-squared test

^b^Mann–Whitney U test

^c^57 lost to follow up

^d^128 lost to follow up

^e^187 lost to follow up

^f^25 lost to follow up

^g^70 lost to follow up

^h^93 lost to follow up

At 6-week follow-up, the following OHS items were significantly different between the two groups (*p* < 0.05), all showing better outcomes in the anterior group (Additional file 1: Appendix Tables): being troubled by pain from the hip in bed at night; sudden, severe pain (shooting, stabbing, or spasms) from affected hip; ability to walk before the pain in the hip becomes severe (with or without a walking aid); ability to climb a flight of stairs and ability to put on a pair of socks, stockings or tights. The breakdown of subgroups for both PROMs can be found as Additional file 1: Appendix 1.

Post-operative scores for all dimensions of the EQ-5D 5L (mobility, self-care, usual activities, pain/discomfort and anxiety/depression) remained similar between both groups at all time points. At 6 weeks, between 175 and 193 (84–93%) DAA patients and 94–101 (86–93%) PA patients had no or slight problems in their EQ-5D 5L dimensions, while between 11 and 25 (5–12%) and between 6 and 11 (6–10%) had moderate pain (*p* > 0.05). At 6 months and 1 year, there was no difference in these scores between both groups either (*p* > 0.05).

The EQ-5D-5L utility scores were also similar pre-operatively between groups [DAA: − 0.030 (IQR − 0.676 to 0.911) and PA: − 0.024 (IQR − 0.176 to 0.367), *p* = 0.47]. At 6 weeks DAA: 0.672 (IQR 0.521–0.805) and PA: 0.672 (IQR 0.502–0.805), *p* = 0.69); 6 months (DAA: 0.860 [IQR 0.661–1.000) and PA: 0.884 (IQR 0.661–1.000), *p* = 0.70] and at 1 year [1.000 points (IQR 0.860–1.000) and 1.000 (IQR 0.733–1.000), respectively, *p* = 0.07] there was no significant difference between groups (Table 3).

**Table 3:** Pre- and post-operative patient-reported outcome measure (PROM) for EQ-5L-5D pain/discomfort score

	Anterior	Posterior	*p* value
EQ-5L-5D pain/discomfort score pre-operatively, *n* (%)^a^	*n* = 337	*n* = 182	
No pain	1 (0)	0 (0)	**0.02**^a^
Slight pain	14 (4)	2 (1)	
Moderate pain	68 (20)	58 (32)	
Severe pain	195 (58)	91 (50)	
Extreme pain	59 (18)	31 (17)	
Lost to follow up	–	–	
EQ-5L-5D pain/discomfort score at 6 weeks, *n* (%)^a^	*n* = 280	*n* = 157	
No pain	63 (23)	46 (29)	0.17^a^
Slight pain	136 (48)	60 (38)	
Moderate pain	67 (24)	44 (28)	
Severe pain	14 (5)	7 (5)	
Extreme pain	0 (0)	0 (0)	
Lost to follow up	57	25	
EQ-5L-5D pain/discomfort score at 6 months, *n* (%)^a^	*n* = 209	*n* = 112	
No pain	112 (54)	64 (57)	0.91^a^
Slight pain	69 (33)	35 (31)	
Moderate pain	22 (11)	10 (9)	
Severe pain	5 (2)	3 (3)	
Extreme pain	1 (0)	0 (0)	
Lost to follow up	128	70	
EQ-5L-5D pain/discomfort score at 1 year, *n* (%)^a^	*n* = 150	*n* = 89	
No pain	119 (79)	63 (71)	0.35^a^
Slight pain	24 (16)	20 (22)	
Moderate pain	6 (4)	5 (6)	
Severe pain	0 (0)	1 (1)	
Extreme pain	1 (1)	0 (0)	
Lost to follow up	187	93	

Bold denotes statistical significance

^a^Chi-squared test

There was no difference in pre- and post-operative anxiety and/or depression scores across both groups (Table 4).

**Table 4:** Pre- and post-operative patient-reported outcome measure (PROM) for EQ-5L-5D anxiety/depression scores

	Anterior	Posterior	*p* value
EQ-5L-5D anxiety/depression score pre-operatively, *n* (%)	*n* = 337	*n* = 182	
Not anxious/depressed	116 (34)	64 (35)	0.65^a^
Slightly anxious/depressed	86 (26)	45 (25)	
Moderately anxious/depressed	109 (32)	53 (29)	
Severely anxious/depressed	14 (4)	13 (7)	
Extremely anxious/depressed	12 (4)	7 (4)	
Lost to follow up	–	–	
EQ-5L-5D anxiety/depression score at 6 weeks, *n* (%)	*n* = 280	*n* = 157	
Not anxious/depressed	199 (71)	113 (72)	0.56^a^
Slightly anxious/depressed	53 (19)	24 (15)	
Moderately anxious/depressed	23 (8)	18 (12)	
Severely anxious/depressed	5 (2)	2 (1)	
Extremely anxious/depressed	0 (0)	0 (0)	
Lost to follow up	57	25	
EQ-5L-5D anxiety/depression score at 6 months, *n* (%)	*n* = 209	*n* = 112	
Not anxious/depressed	156 (75)	90 (80)	0.62^a^
Slightly anxious/depressed	38 (18)	14 (13)	
Moderately anxious/depressed	11 (5)	6 (5)	
Severely anxious/depressed	4 (2)	2 (2)	
Extremely anxious/depressed	0 (0)	0 (0)	
Lost to follow up	128	70	
EQ-5L-5D anxiety/depression score at 1 year, *n* (%)	*n* = 150	*n* = 89	
Not anxious/depressed	121 (81)	71 (80)	0.97^a^
Slightly anxious/depressed	22 (15)	13 (15)	
Moderately anxious/depressed	5 (3)	4 (4)	
Severely anxious/depressed	2 (1)	1 (1)	
Extremely anxious/depressed	0 (0)	0 (0)	
Lost to follow up	187	93	

^a^Chi-squared test

Patient satisfaction scores were also similar between both groups at all time points (Table 5).

**Table 5:** Post-operative patient satisfaction

	Anterior	Posterior	*p* value
Patient satisfaction at 6 weeks, *n* (%)	*n* = 280	*n* = 157	
Very satisfied	176 (63)	103 (66)	0.66^a^
Satisfied	80 (28)	39 (25)	
Ambivalent	8 (3)	7 (4)	
Dissatisfied	3 (1)	3 (2)	
Very dissatisfied	13 (5)	5 (3)	
Lost to follow up	57	25	
Patient satisfaction at 6 months, *n* (%)	*n* = 209	*n* = 112	
Very satisfied	135 (65)	79 (71)	0.69^a^
Satisfied	52 (25)	25 (22)	
Ambivalent	10 (5)	4 (4)	
Dissatisfied	3 (1)	2 (1.5)	
Very dissatisfied	9 (4)	2 (1.5)	
Lost to follow up	128	70	
Patient satisfaction at 1 year, *n* (%)	*n* = 150	*n* = 89	
Very satisfied	102 (68)	62 (70)	0.56^a^
Satisfied	34 (23)	21 (24)	
Ambivalent	5 (3)	4 (4)	
Dissatisfied	1 (1)	1 (1)	
Very dissatisfied	8 (5)	1 (1)	
Lost to follow up	187	93	

^a^Chi-squared test

## Discussion

The aim of this prospective multi-centre trial was to determine if there was a difference between the direct anterior approach and the posterior approach for total hip arthroplasty surgery in terms of length of stay, long-term functional recovery and pain scores.

This prospective multi-centre study found that patients undergoing a direct anterior approach for total hip arthroplasty report improved quality of recovery with shorter LOS and better PROMs at 6 weeks post-operatively compared to patients undergoing a posterior approach. There are no long-term benefits between both surgical approaches. Given the recovery trajectory is that patients are usually fully recovered well before 6 months, there is also no reason to expect a difference at this time point. Previous studies have also demonstrated an improvement in functional outcomes and shorter LOS with the DAA, with a consequent reduction in post-operative complications [14]. This is consistent with the less invasive approach of the DAA which utilises anatomical tissue planes.

The Oxford Hip Score and EQ-5D-5L Health Questionnaire are validated PROMs, widely used to assess joint related disability and functional recovery in orthopaedic surgery [15, 16]. Internal consistency is high for both questionnaires; Cronbach alpha = 0.94 for the Oxford Hip Score, [17] and 0.86 for the EQ-5D-5L [18]. In the current study, the OHS showed an early improvement in favour of DAA patients at 6 weeks. The EQ-5D-5L showed no difference between the two groups at any time point. At 6 months and at 1 year, there was no difference in functional recovery between the two groups. A reason for the EQ-5D-5L not being different between groups while the OHS was, is that the OHS is a joint-specific PROM while the EQ-5D-5L measures general health. It may thus be that the OHS can detect more joint-specific improvement, whereas the EQ-5D-5L is less specific in this regard. Hence, these results imply that both groups were similar in overall general health, but that the early OHS improvement in the DAA group reflects a better early functional recovery for patients undergoing DAA.

Previous analyses have also reported superior early recovery of DAA compared to PA, which is likely due to the minimally invasive nature of the technique. Most of these studies, however, have been limited in their duration of follow-up, of low quality, have not formally assessed functionality using a validated PROM or have not achieved minimally clinical important difference in the PROMs [19]. Previous studies that have reported long-term outcomes have also not been able to show a benefit of either technique [20]. In the current study, we confirm these outcomes, but now with a prospective cohort design, and systematic use of PROMs.

Earlier studies have often focussed on gait analysis, radiographic outcomes, dislocation rates or length of stay as primary end points. We aimed to assess global recovery and functionality utilising LOS, and the Oxford Hip Score and the EQ-5D-5L questionnaires. There is a training curve for the DAA approach, which represents a significant investment for both the surgeon and the patients involved. It is also well recognised that certain patient types lend themselves better to the DAA approach, such as the non-obese patient for example. Hence, careful patient selection and a risk–benefit analysis must form a part of the consideration for each individual surgeon when choosing a surgical approach.

Some limitations of the current study have to be addressed. Seventeen per cent of patients were lost to follow up at 6 weeks, 38% at 6 months and 64% at 1 year. Similar losses to follow up at similar time points were reported in previous studies of THA outcomes [21]. Baseline characteristics between both groups were not balanced as the DAA group had a lower BMI, a greater proportion of females, and had more pre-operative pain. This represents a selection bias, as these characteristics make the patient more suitable for the DAA approach. Due to the team-based approach to patient selection for surgery and allocation to individual surgeons who favour one approach above the other, this was a conscious decision made to optimise patient outcome and allow surgeons training in the DAA the most optimal conditions in which to begin. That this study suggests favourable outcomes for the DAA compared to PA, despite the inclusion of training data, speaks to the possible short-term benefits of this technique [22, 23]. It is also dubious if a BMI difference between the two groups of 1.7 kg/m^2^ would be clinically significant, despite the statistical significance. The gender imbalance between the groups, with more female patients undergoing DAA, does raise an interesting point. In previously published literature, patients with a female gender have been described as having a poorer post-operative trajectory including a significantly longer length of stay than their male counterparts (more than 2 days longer per length of stay (*p* < 0.0001) [24] That a female heavy group in our study had a shorter length of stay is perhaps more impressive in this context.

In conclusion, the results of this multi-centre prospective study complement previous studies showing early functional improvement in favour of the DAA approach. However, there is no difference in long-term PROM outcomes, pain scores or patient satisfaction between the two approaches. Future direction for investigation should include well-designed multi-centre randomised controlled trials to compare long-term effects of both approaches.

## Supplementary Information

**Additional file 1.** Additional statistical analyses.

## Abbreviations

DAA: Direct anterior approach
THA: Total hip arthroplasty
PA: Posterior approach
PROMs: Patient-reported outcome measures
OHS: Oxford Hip Score
LOS: Length of stay
BMI: Body mass index
