# Supplementary material for: Short-term difference only in reported outcomes (PROMs) after anterior or posterior approach to total hip arthroplasty: a 4-year prospective multi-centre observational study
Source: J Orthop Surg Res. 2023 Feb 17;18:119. doi: 10.1186/s13018-023-03603-0 (PMC9936928; doi:10.1186/s13018-023-03603-0)
Supplement: Supplementary file 1 — Additional file 1. Additional statistical analyses. [file 13018_2023_3603_MOESM1_ESM.docx]

**Appendix tables:**

***Tabulation of subgroups of EQ-5D-5L as frequencies and percentages***

**Tabulation of EQ5D5L_Mobility Group when TIMEPOINT is Pre-operatively**

| Mobility | Group | | |
| --- | --- | --- | --- |
|  | Anterior | Posterior | Total |
| No problems walking about | 126 | 63 | 189 |
|  | 82.89 | 68.48 | 77.46 |
| Slight problems walking about | 20 | 24 | 44 |
|  | 13.16 | 26.09 | 18.03 |
| Moderate problems walking about | 5 | 4 | 9 |
|  | 3.29 | 4.35 | 3.69 |
| Severe problems walking about | 1 | 0 | 1 |
|  | 0.66 | 0.00 | 0.41 |
| Unable to walk about | 0 | 1 | 1 |
|  | 0.00 | 1.09 | 0.41 |
| Total | 152 | 92 | 244 |
|  | 100.00 | 100.00 | 100.00 |
| Pearson Chi2 = 9.28 Prob = 0.0544 | | | |

First row has *frequencies* and second row has *column percentages*

**Tabulation of EQ5D5L_Mobility Group when TIMEPOINT is 6-weeks**

| Mobility | Group | | |
| --- | --- | --- | --- |
|  | Anterior | Posterior | Total |
| No problems walking about | 119 | 57 | 176 |
|  | 57.21 | 52.29 | 55.52 |
| Slight problems walking about | 60 | 39 | 99 |
|  | 28.85 | 35.78 | 31.23 |
| Moderate problems walking about | 23 | 11 | 34 |
|  | 11.06 | 10.09 | 10.73 |
| Severe problems walking about | 6 | 2 | 8 |
|  | 2.88 | 1.83 | 2.52 |
| Total | 208 | 109 | 317 |
|  | 100.00 | 100.00 | 100.00 |
| Pearson Chi2 = 1.79 Prob = 0.6178 | | | |

First row has *frequencies* and second row has *column percentages*

**Tabulation of EQ5D5L_Mobility Group when TIMEPOINT is 6-months**

| Mobility | Group | | |
| --- | --- | --- | --- |
|  | Anterior | Posterior | Total |
| No problems walking about | 83 | 44 | 127 |
|  | 30.29 | 28.39 | 29.60 |
| Slight problems walking about | 102 | 51 | 153 |
|  | 37.23 | 32.90 | 35.66 |
| Moderate problems walking about | 77 | 47 | 124 |
|  | 28.10 | 30.32 | 28.90 |
| Severe problems walking about | 12 | 13 | 25 |
|  | 4.38 | 8.39 | 5.83 |
| Total | 274 | 155 | 429 |
|  | 100.00 | 100.00 | 100.00 |
| Pearson Chi2 = 3.54 Prob = 0.3160 | | | |

First row has *frequencies* and second row has *column percentages*

**Tabulation of EQ5D5L_Mobility Group when TIMEPOINT is 1-year**

| Mobility | Group | | |
| --- | --- | --- | --- |
|  | Anterior | Posterior | Total |
| No problems walking about | 5 | 2 | 7 |
|  | 1.74 | 1.20 | 1.54 |
| Slight problems walking about | 18 | 10 | 28 |
|  | 6.25 | 6.02 | 6.17 |
| Moderate problems walking about | 74 | 38 | 112 |
|  | 25.69 | 22.89 | 24.67 |
| Severe problems walking about | 174 | 98 | 272 |
|  | 60.42 | 59.04 | 59.91 |
| Unable to walk about | 17 | 18 | 35 |
|  | 5.90 | 10.84 | 7.71 |
| Total | 288 | 166 | 454 |
|  | 100.00 | 100.00 | 100.00 |
| Pearson Chi2 = 3.90 Prob = 0.4191 | | | |

First row has *frequencies* and second row has *column percentages*

**Tabulation of EQ5D5L_Selfcare Group when TIMEPOINT is Pre-operatively**

| Self-care | Group | | |
| --- | --- | --- | --- |
|  | Anterior | Posterior | Total |
| No problems | 129 | 71 | 200 |
|  | 84.87 | 77.17 | 81.97 |
| Slight problems | 15 | 16 | 31 |
|  | 9.87 | 17.39 | 12.70 |
| Moderate problems | 8 | 4 | 12 |
|  | 5.26 | 4.35 | 4.92 |
| Unable to peform | 0 | 1 | 1 |
|  | 0.00 | 1.09 | 0.41 |
| Total | 152 | 92 | 244 |
|  | 100.00 | 100.00 | 100.00 |
| Pearson Chi2 = 4.72 Prob = 0.1938 | | | |

First row has *frequencies* and second row has *column percentages*

**Tabulation of EQ5D5L_Selfcare Group when TIMEPOINT is 6-weeks**

| Self-care | Group | | |
| --- | --- | --- | --- |
|  | Anterior | Posterior | Total |
| No problems | 140 | 74 | 214 |
|  | 67.31 | 67.89 | 67.51 |
| Slight problems | 46 | 26 | 72 |
|  | 22.12 | 23.85 | 22.71 |
| Moderate problems | 17 | 7 | 24 |
|  | 8.17 | 6.42 | 7.57 |
| Severe problems | 4 | 2 | 6 |
|  | 1.92 | 1.83 | 1.89 |
| Unable to perform | 1 | 0 | 1 |
|  | 0.48 | 0.00 | 0.32 |
| Total | 208 | 109 | 317 |
|  | 100.00 | 100.00 | 100.00 |
| Pearson Chi2 = 0.92 Prob = 0.9224 | | | |

First row has *frequencies* and second row has *column percentages*

**Tabulation of EQ5D5L_Selfcare Group when TIMEPOINT is 6-months**

| Self-care | Group | | |
| --- | --- | --- | --- |
|  | Anterior | Posterior | Total |
| No problems | 137 | 81 | 218 |
|  | 50.00 | 52.26 | 50.82 |
| Slight problems | 84 | 45 | 129 |
|  | 30.66 | 29.03 | 30.07 |
| Moderate problems | 45 | 20 | 65 |
|  | 16.42 | 12.90 | 15.15 |
| Severe problems | 7 | 7 | 14 |
|  | 2.55 | 4.52 | 3.26 |
| Unable to perform | 1 | 2 | 3 |
|  | 0.36 | 1.29 | 0.70 |
| Total | 274 | 155 | 429 |
|  | 100.00 | 100.00 | 100.00 |
| Pearson Chi2 = 3.38 Prob = 0.4971 | | | |

First row has *frequencies* and second row has *column percentages*

**Tabulation of EQ5D5L_Selfcare Group when TIMEPOINT is 1-year**

| Self-care | Group | | |
| --- | --- | --- | --- |
|  | Anterior | Posterior | Total |
| No problems | 46 | 34 | 80 |
|  | 15.97 | 20.48 | 17.62 |
| Slight problems | 73 | 29 | 102 |
|  | 25.35 | 17.47 | 22.47 |
| Moderate problems | 75 | 48 | 123 |
|  | 26.04 | 28.92 | 27.09 |
| Severe problems | 87 | 46 | 133 |
|  | 30.21 | 27.71 | 29.30 |
| Unable to perform | 7 | 9 | 16 |
|  | 2.43 | 5.42 | 3.52 |
| Total | 288 | 166 | 454 |
|  | 100.00 | 100.00 | 100.00 |
| Pearson Chi2 = 7.34 Prob = 0.1189 | | | |

First row has *frequencies* and second row has *column percentages*

**Tabulation of EQ5D5L_UsualActivities Group when TIMEPOINT is Pre-operatively**

| Usual activities | Group | | |
| --- | --- | --- | --- |
|  | Anterior | Posterior | Total |
| No problems | 123 | 66 | 189 |
|  | 80.92 | 71.74 | 77.46 |
| Slight problems | 20 | 18 | 38 |
|  | 13.16 | 19.57 | 15.57 |
| Moderate problems | 8 | 7 | 15 |
|  | 5.26 | 7.61 | 6.15 |
| Severe problems | 1 | 0 | 1 |
|  | 0.66 | 0.00 | 0.41 |
| Unable to perform | 0 | 1 | 1 |
|  | 0.00 | 1.09 | 0.41 |
| Total | 152 | 92 | 244 |
|  | 100.00 | 100.00 | 100.00 |
| Pearson Chi2 = 4.90 Prob = 0.2972 | | | |

First row has *frequencies* and second row has *column percentages*

**Tabulation of EQ5D5L_UsualActivities Group when TIMEPOINT is 6-weeks**

| Usual activities | Group | | |
| --- | --- | --- | --- |
|  | Anterior | Posterior | Total |
| No problems | 118 | 59 | 177 |
|  | 56.73 | 54.13 | 55.84 |
| Slight problems | 57 | 35 | 92 |
|  | 27.40 | 32.11 | 29.02 |
| Moderate problems | 25 | 9 | 34 |
|  | 12.02 | 8.26 | 10.73 |
| Severe problems | 7 | 6 | 13 |
|  | 3.37 | 5.50 | 4.10 |
| Unable to perform | 1 | 0 | 1 |
|  | 0.48 | 0.00 | 0.32 |
| Total | 208 | 109 | 317 |
|  | 100.00 | 100.00 | 100.00 |
| Pearson Chi2 = 2.90 Prob = 0.5749 | | | |

First row has *frequencies* and second row has *column percentages*

**Tabulation of EQ5D5L_UsualActivities Group when TIMEPOINT is 6-months**

| Usual activities | Group | | |
| --- | --- | --- | --- |
|  | Anterior | Posterior | Total |
| No problems | 69 | 39 | 108 |
|  | 25.18 | 25.16 | 25.17 |
| Slight problems | 113 | 51 | 164 |
|  | 41.24 | 32.90 | 38.23 |
| Moderate problems | 69 | 43 | 112 |
|  | 25.18 | 27.74 | 26.11 |
| Severe problems | 17 | 16 | 33 |
|  | 6.20 | 10.32 | 7.69 |
| Unable to perform | 6 | 6 | 12 |
|  | 2.19 | 3.87 | 2.80 |
| Total | 274 | 155 | 429 |
|  | 100.00 | 100.00 | 100.00 |
| Pearson Chi2 = 5.23 Prob = 0.2643 | | | |

First row has *frequencies* and second row has *column percentages*

**Tabulation of EQ5D5L_UsualActivities Group when TIMEPOINT is 1-year**

| Usual activities | Group | | |
| --- | --- | --- | --- |
|  | Anterior | Posterior | Total |
| No problems | 8 | 3 | 11 |
|  | 2.78 | 1.81 | 2.42 |
| Slight problems | 23 | 12 | 35 |
|  | 7.99 | 7.23 | 7.71 |
| Moderate problems | 81 | 41 | 122 |
|  | 28.12 | 24.70 | 26.87 |
| Severe problems | 139 | 83 | 222 |
|  | 48.26 | 50.00 | 48.90 |
| Unable to perform | 37 | 27 | 64 |
|  | 12.85 | 16.27 | 14.10 |
| Total | 288 | 166 | 454 |
|  | 100.00 | 100.00 | 100.00 |
| Pearson Chi2 = 1.89 Prob = 0.7569 | | | |

First row has *frequencies* and second row has *column percentages*

**Tabulation of EQ5D5L_Pain_Discomfort Group when TIMEPOINT is Pre-operatively**

| Pain/Discomfort | Group | | |
| --- | --- | --- | --- |
|  | Anterior | Posterior | Total |
| No problems | 122 | 65 | 187 |
|  | 80.26 | 70.65 | 76.64 |
| Slight problems | 23 | 21 | 44 |
|  | 15.13 | 22.83 | 18.03 |
| Moderate problems | 6 | 5 | 11 |
|  | 3.95 | 5.43 | 4.51 |
| Severe problems | 0 | 1 | 1 |
|  | 0.00 | 1.09 | 0.41 |
| Unable to perform | 1 | 0 | 1 |
|  | 0.66 | 0.00 | 0.41 |
| Total | 152 | 92 | 244 |
|  | 100.00 | 100.00 | 100.00 |
| Pearson Chi2 = 5.11 Prob = 0.2761 | | | |

First row has *frequencies* and second row has *column percentages*

**Tabulation of EQ5D5L_Pain_Discomfort Group when TIMEPOINT is 6-weeks**

| Pain/Discomfort | Group | | |
| --- | --- | --- | --- |
|  | Anterior | Posterior | Total |
| No problems walking about | 110 | 63 | 173 |
|  | 52.88 | 57.80 | 54.57 |
| Slight problems walking about | 70 | 33 | 103 |
|  | 33.65 | 30.28 | 32.49 |
| Moderate problems walking about | 22 | 10 | 32 |
|  | 10.58 | 9.17 | 10.09 |
| Severe problems walking about | 5 | 3 | 8 |
|  | 2.40 | 2.75 | 2.52 |
| Unable to walk about | 1 | 0 | 1 |
|  | 0.48 | 0.00 | 0.32 |
| Total | 208 | 109 | 317 |
|  | 100.00 | 100.00 | 100.00 |
| Pearson Chi2 = 1.27 Prob = 0.8672 | | | |

First row has *frequencies* and second row has *column percentages*

**Tabulation of EQ5D5L_Pain_Discomfort Group when TIMEPOINT is 6-months**

| Pain/Discomfort | Group | | |
| --- | --- | --- | --- |
|  | Anterior | Posterior | Total |
| No problems walking about | 62 | 45 | 107 |
|  | 22.63 | 29.03 | 24.94 |
| Slight problems walking about | 133 | 60 | 193 |
|  | 48.54 | 38.71 | 44.99 |
| Moderate problems walking about | 65 | 43 | 108 |
|  | 23.72 | 27.74 | 25.17 |
| Severe problems walking about | 14 | 7 | 21 |
|  | 5.11 | 4.52 | 4.90 |
| Total | 274 | 155 | 429 |
|  | 100.00 | 100.00 | 100.00 |
| Pearson Chi2 = 4.46 Prob = 0.2158 | | | |

First row has *frequencies* and second row has *column percentages*

**Tabulation of EQ5D5L_Pain_Discomfort Group when TIMEPOINT is 1-year**

| Pain/Discomfort | Group | | |
| --- | --- | --- | --- |
|  | Anterior | Posterior | Total |
| No problems walking about | 1 | 0 | 1 |
|  | 0.35 | 0.00 | 0.22 |
| Slight problems walking about | 14 | 2 | 16 |
|  | 4.86 | 1.20 | 3.52 |
| Moderate problems walking about | 68 | 42 | 110 |
|  | 23.61 | 25.30 | 24.23 |
| Severe problems walking about | 151 | 90 | 241 |
|  | 52.43 | 54.22 | 53.08 |
| Unable to walk about | 54 | 32 | 86 |
|  | 18.75 | 19.28 | 18.94 |
| Total | 288 | 166 | 454 |
|  | 100.00 | 100.00 | 100.00 |
| Pearson Chi2 = 4.77 Prob = 0.3113 | | | |

First row has *frequencies* and second row has *column percentages*

**Tabulation of EQ5D5L_Anxiety_Depression Group when TIMEPOINT is Pre-operatively**

| Anxiety/Depression | Group | | |
| --- | --- | --- | --- |
|  | Anterior | Posterior | Total |
| No problems | 122 | 73 | 195 |
|  | 80.26 | 79.35 | 79.92 |
| Slight problems | 23 | 13 | 36 |
|  | 15.13 | 14.13 | 14.75 |
| Moderate problems | 5 | 5 | 10 |
|  | 3.29 | 5.43 | 4.10 |
| Severe problems | 2 | 1 | 3 |
|  | 1.32 | 1.09 | 1.23 |
| Total | 152 | 92 | 244 |
|  | 100.00 | 100.00 | 100.00 |
| Pearson Chi2 = 0.71 Prob = 0.8702 | | | |

First row has *frequencies* and second row has *column percentages*

**Tabulation of EQ5D5L_Anxiety_Depression Group when TIMEPOINT is 6-weeks**

| Anxiety/Depression | Group | | |
| --- | --- | --- | --- |
|  | Anterior | Posterior | Total |
| No problems | 155 | 87 | 242 |
|  | 74.52 | 79.82 | 76.34 |
| Slight problems | 38 | 14 | 52 |
|  | 18.27 | 12.84 | 16.40 |
| Moderate problems | 11 | 6 | 17 |
|  | 5.29 | 5.50 | 5.36 |
| Severe problems | 4 | 2 | 6 |
|  | 1.92 | 1.83 | 1.89 |
| Total | 208 | 109 | 317 |
|  | 100.00 | 100.00 | 100.00 |
| Pearson Chi2 = 1.56 Prob = 0.6696 | | | |

First row has *frequencies* and second row has *column percentages*

**Tabulation of EQ5D5L_Anxiety_Depression Group when TIMEPOINT is 6-months**

| Anxiety/Depression | Group | | |
| --- | --- | --- | --- |
|  | Anterior | Posterior | Total |
| No problems | 194 | 112 | 306 |
|  | 70.80 | 72.26 | 71.33 |
| Slight problems | 52 | 24 | 76 |
|  | 18.98 | 15.48 | 17.72 |
| Moderate problems | 23 | 17 | 40 |
|  | 8.39 | 10.97 | 9.32 |
| Severe problems | 5 | 2 | 7 |
|  | 1.82 | 1.29 | 1.63 |
| Total | 274 | 155 | 429 |
|  | 100.00 | 100.00 | 100.00 |
| Pearson Chi2 = 1.59 Prob = 0.6621 | | | |

First row has *frequencies* and second row has *column percentages*

**Tabulation of EQ5D5L_Anxiety_Depression Group when TIMEPOINT is 1-year**

| Anxiety/Depression | Group | | |
| --- | --- | --- | --- |
|  | Anterior | Posterior | Total |
| No problems | 117 | 62 | 179 |
|  | 40.62 | 37.35 | 39.43 |
| Slight problems | 85 | 46 | 131 |
|  | 29.51 | 27.71 | 28.85 |
| Moderate problems | 59 | 38 | 97 |
|  | 20.49 | 22.89 | 21.37 |
| Severe problems | 15 | 13 | 28 |
|  | 5.21 | 7.83 | 6.17 |
| Unable to function | 12 | 7 | 19 |
|  | 4.17 | 4.22 | 4.19 |
| Total | 288 | 166 | 454 |
|  | 100.00 | 100.00 | 100.00 |
| Pearson Chi2 = 1.87 Prob = 0.7604 | | | |

First row has *frequencies* and second row has *column percentages*

***Tabulation of subgroups of Oxford Hip Score as frequencies and percentages***

**Tabulation of UsualPain Group when TIMEPOINT is Pre-operatively**

| UsualPain | Group | | |
| --- | --- | --- | --- |
|  | Anterior | Posterior | Total |
| Severe | 93 | 41 | 134 |
|  | 37.80 | 30.83 | 35.36 |
| Moderate | 3 | 2 | 5 |
|  | 1.22 | 1.50 | 1.32 |
| Mild | 6 | 5 | 11 |
|  | 2.44 | 3.76 | 2.90 |
| Very mild | 23 | 19 | 42 |
|  | 9.35 | 14.29 | 11.08 |
| None | 121 | 66 | 187 |
|  | 49.19 | 49.62 | 49.34 |
| Total | 246 | 133 | 379 |
|  | 100.00 | 100.00 | 100.00 |
| Pearson Chi2 = 3.66 Prob = 0.4537 | | | |

First row has *frequencies* and second row has *column percentages*

**Tabulation of UsualPain Group when TIMEPOINT is 6-weeks**

| UsualPain | Group | | |
| --- | --- | --- | --- |
|  | Anterior | Posterior | Total |
| Severe | 62 | 46 | 108 |
|  | 23.05 | 29.49 | 25.41 |
| Moderate | 11 | 4 | 15 |
|  | 4.09 | 2.56 | 3.53 |
| Mild | 19 | 6 | 25 |
|  | 7.06 | 3.85 | 5.88 |
| Very mild | 80 | 55 | 135 |
|  | 29.74 | 35.26 | 31.76 |
| None | 97 | 45 | 142 |
|  | 36.06 | 28.85 | 33.41 |
| Total | 269 | 156 | 425 |
|  | 100.00 | 100.00 | 100.00 |
| Pearson Chi2 = 6.48 Prob = 0.1659 | | | |

First row has *frequencies* and second row has *column percentages*

**Tabulation of UsualPain Group when TIMEPOINT is 6-months**

| UsualPain | Group | | |
| --- | --- | --- | --- |
|  | Anterior | Posterior | Total |
| Severe | 29 | 19 | 48 |
|  | 9.57 | 11.18 | 10.15 |
| Moderate | 42 | 22 | 64 |
|  | 13.86 | 12.94 | 13.53 |
| Mild | 96 | 44 | 140 |
|  | 31.68 | 25.88 | 29.60 |
| Very mild | 87 | 57 | 144 |
|  | 28.71 | 33.53 | 30.44 |
| None | 49 | 28 | 77 |
|  | 16.17 | 16.47 | 16.28 |
| Total | 303 | 170 | 473 |
|  | 100.00 | 100.00 | 100.00 |
| Pearson Chi2 = 2.42 Prob = 0.6593 | | | |

First row has *frequencies* and second row has *column percentages*

**Tabulation of UsualPain Group when TIMEPOINT is 1-year**

| UsualPain | Group | | |
| --- | --- | --- | --- |
|  | Anterior | Posterior | Total |
| Severe | 189 | 101 | 290 |
|  | 55.92 | 55.19 | 55.66 |
| Moderate | 121 | 65 | 186 |
|  | 35.80 | 35.52 | 35.70 |
| Mild | 23 | 14 | 37 |
|  | 6.80 | 7.65 | 7.10 |
| Very mild | 3 | 2 | 5 |
|  | 0.89 | 1.09 | 0.96 |
| 5 | 2 | 1 | 3 |
|  | 0.59 | 0.55 | 0.58 |
| Total | 338 | 183 | 521 |
|  | 100.00 | 100.00 | 100.00 |
| Pearson Chi2 = 0.19 Prob = 0.9958 | | | |

First row has *frequencies* and second row has *column percentages*

**Tabulation of PainAtNight Group when TIMEPOINT is Pre-operatively**

| PainAtNight | Group | | |
| --- | --- | --- | --- |
|  | Anterior | Posterior | Total |
| Every night | 93 | 41 | 134 |
|  | 37.80 | 30.83 | 35.36 |
| Most nights | 0 | 1 | 1 |
|  | 0.00 | 0.75 | 0.26 |
| Some nights | 8 | 6 | 14 |
|  | 3.25 | 4.51 | 3.69 |
| Only 1 or 2 nights | 16 | 15 | 31 |
|  | 6.50 | 11.28 | 8.18 |
| No nights | 129 | 70 | 199 |
|  | 52.44 | 52.63 | 52.51 |
| Total | 246 | 133 | 379 |
|  | 100.00 | 100.00 | 100.00 |
| Pearson Chi2 = 5.82 Prob = 0.2134 | | | |

First row has *frequencies* and second row has *column percentages*

**Tabulation of PainAtNight Group when TIMEPOINT is 6-weeks**

| PainAtNight | Group | | |
| --- | --- | --- | --- |
|  | Anterior | Posterior | Total |
| Every night | 61 | 48 | 109 |
|  | 22.68 | 30.77 | 25.65 |
| Most nights | 1 | 1 | 2 |
|  | 0.37 | 0.64 | 0.47 |
| Some nights | 26 | 4 | 30 |
|  | 9.67 | 2.56 | 7.06 |
| Only 1 or 2 nights | 54 | 58 | 112 |
|  | 20.07 | 37.18 | 26.35 |
| No nights | 127 | 45 | 172 |
|  | 47.21 | 28.85 | 40.47 |
| Total | 269 | 156 | 425 |
|  | 100.00 | 100.00 | 100.00 |
| Pearson Chi2 = 28.92 Prob = 0.0000 | | | |

First row has *frequencies* and second row has *column percentages*

**Tabulation of PainAtNight Group when TIMEPOINT is 6-months**

| PainAtNight | Group | | |
| --- | --- | --- | --- |
|  | Anterior | Posterior | Total |
| Every night | 26 | 14 | 40 |
|  | 8.58 | 8.24 | 8.46 |
| Most nights | 7 | 3 | 10 |
|  | 2.31 | 1.76 | 2.11 |
| Some nights | 70 | 53 | 123 |
|  | 23.10 | 31.18 | 26.00 |
| Only 1 or 2 nights | 120 | 48 | 168 |
|  | 39.60 | 28.24 | 35.52 |
| No nights | 80 | 52 | 132 |
|  | 26.40 | 30.59 | 27.91 |
| Total | 303 | 170 | 473 |
|  | 100.00 | 100.00 | 100.00 |
| Pearson Chi2 = 7.55 Prob = 0.1097 | | | |

First row has *frequencies* and second row has *column percentages*

**Tabulation of PainAtNight Group when TIMEPOINT is 1-year**

| PainAtNight | Group | | |
| --- | --- | --- | --- |
|  | Anterior | Posterior | Total |
| Every night | 63 | 32 | 95 |
|  | 18.64 | 17.49 | 18.23 |
| Most nights | 103 | 51 | 154 |
|  | 30.47 | 27.87 | 29.56 |
| Some nights | 112 | 73 | 185 |
|  | 33.14 | 39.89 | 35.51 |
| Only 1 or 2 nights | 49 | 13 | 62 |
|  | 14.50 | 7.10 | 11.90 |
| No nights | 8 | 13 | 21 |
|  | 2.37 | 7.10 | 4.03 |
| 5 | 3 | 1 | 4 |
|  | 0.89 | 0.55 | 0.77 |
| Total | 338 | 183 | 521 |
|  | 100.00 | 100.00 | 100.00 |
| Pearson Chi2 = 14.13 Prob = 0.0148 | | | |

First row has *frequencies* and second row has *column percentages*

**Tabulation of SuddenPain Group when TIMEPOINT is Pre-operatively**

| SuddenPain | Group | | |
| --- | --- | --- | --- |
|  | Anterior | Posterior | Total |
| Every day | 93 | 41 | 134 |
|  | 37.80 | 30.83 | 35.36 |
| Most days | 1 | 2 | 3 |
|  | 0.41 | 1.50 | 0.79 |
| Some days | 9 | 7 | 16 |
|  | 3.66 | 5.26 | 4.22 |
| Only 1 or 2 days | 22 | 20 | 42 |
|  | 8.94 | 15.04 | 11.08 |
| No days | 121 | 63 | 184 |
|  | 49.19 | 47.37 | 48.55 |
| Total | 246 | 133 | 379 |
|  | 100.00 | 100.00 | 100.00 |
| Pearson Chi2 = 5.98 Prob = 0.2006 | | | |

First row has *frequencies* and second row has *column percentages*

**Tabulation of SuddenPain Group when TIMEPOINT is 6-weeks**

| SuddenPain | Group | | |
| --- | --- | --- | --- |
|  | Anterior | Posterior | Total |
| Every day | 60 | 46 | 106 |
|  | 22.30 | 29.49 | 24.94 |
| Most days | 3 | 4 | 7 |
|  | 1.12 | 2.56 | 1.65 |
| Some days | 29 | 10 | 39 |
|  | 10.78 | 6.41 | 9.18 |
| Only 1 or 2 days | 77 | 59 | 136 |
|  | 28.62 | 37.82 | 32.00 |
| No days | 100 | 37 | 137 |
|  | 37.17 | 23.72 | 32.24 |
| Total | 269 | 156 | 425 |
|  | 100.00 | 100.00 | 100.00 |
| Pearson Chi2 = 13.51 Prob = 0.0090 | | | |

First row has *frequencies* and second row has *column percentages*

**Tabulation of SuddenPain Group when TIMEPOINT is 6-months**

| SuddenPain | Group | | |
| --- | --- | --- | --- |
|  | Anterior | Posterior | Total |
| Every day | 25 | 14 | 39 |
|  | 8.25 | 8.24 | 8.25 |
| Most days | 12 | 17 | 29 |
|  | 3.96 | 10.00 | 6.13 |
| Some days | 82 | 51 | 133 |
|  | 27.06 | 30.00 | 28.12 |
| Only 1 or 2 days | 117 | 51 | 168 |
|  | 38.61 | 30.00 | 35.52 |
| No days | 67 | 37 | 104 |
|  | 22.11 | 21.76 | 21.99 |
| Total | 303 | 170 | 473 |
|  | 100.00 | 100.00 | 100.00 |
| Pearson Chi2 = 9.09 Prob = 0.0588 | | | |

First row has *frequencies* and second row has *column percentages*

**Tabulation of SuddenPain Group when TIMEPOINT is 1-year**

| SuddenPain | Group | | |
| --- | --- | --- | --- |
|  | Anterior | Posterior | Total |
| Every day | 51 | 26 | 77 |
|  | 15.09 | 14.21 | 14.78 |
| Most days | 144 | 91 | 235 |
|  | 42.60 | 49.73 | 45.11 |
| Some days | 116 | 51 | 167 |
|  | 34.32 | 27.87 | 32.05 |
| Only 1 or 2 days | 23 | 9 | 32 |
|  | 6.80 | 4.92 | 6.14 |
| No days | 2 | 5 | 7 |
|  | 0.59 | 2.73 | 1.34 |
| 5 | 2 | 1 | 3 |
|  | 0.59 | 0.55 | 0.58 |
| Total | 338 | 183 | 521 |
|  | 100.00 | 100.00 | 100.00 |
| Pearson Chi2 = 7.68 Prob = 0.1748 | | | |

First row has *frequencies* and second row has *column percentages*

**Tabulation of Limping Group when TIMEPOINT is Pre-operatively**

| Limping | Group | | |
| --- | --- | --- | --- |
|  | Anterior | Posterior | Total |
| All of the time | 100 | 48 | 148 |
|  | 40.65 | 36.09 | 39.05 |
| Most of the time | 3 | 3 | 6 |
|  | 1.22 | 2.26 | 1.58 |
| Often | 16 | 8 | 24 |
|  | 6.50 | 6.02 | 6.33 |
| Sometimes | 28 | 20 | 48 |
|  | 11.38 | 15.04 | 12.66 |
| Rarely/never | 99 | 54 | 153 |
|  | 40.24 | 40.60 | 40.37 |
| Total | 246 | 133 | 379 |
|  | 100.00 | 100.00 | 100.00 |
| Pearson Chi2 = 1.99 Prob = 0.7374 | | | |

First row has *frequencies* and second row has *column percentages*

**Tabulation of Limping Group when TIMEPOINT is 6-weeks**

| Limping | Group | | |
| --- | --- | --- | --- |
|  | Anterior | Posterior | Total |
| All of the time | 77 | 56 | 133 |
|  | 28.62 | 35.90 | 31.29 |
| Most of the time | 15 | 4 | 19 |
|  | 5.58 | 2.56 | 4.47 |
| Often | 29 | 15 | 44 |
|  | 10.78 | 9.62 | 10.35 |
| Sometimes | 79 | 56 | 135 |
|  | 29.37 | 35.90 | 31.76 |
| Rarely/never | 69 | 25 | 94 |
|  | 25.65 | 16.03 | 22.12 |
| Total | 269 | 156 | 425 |
|  | 100.00 | 100.00 | 100.00 |
| Pearson Chi2 = 9.26 Prob = 0.0548 | | | |

First row has *frequencies* and second row has *column percentages*

**Tabulation of Limping Group when TIMEPOINT is 6-months**

| Limping | Group | | |
| --- | --- | --- | --- |
|  | Anterior | Posterior | Total |
| All of the time | 73 | 50 | 123 |
|  | 24.09 | 29.41 | 26.00 |
| Most of the time | 24 | 20 | 44 |
|  | 7.92 | 11.76 | 9.30 |
| Often | 94 | 38 | 132 |
|  | 31.02 | 22.35 | 27.91 |
| Sometimes | 75 | 40 | 115 |
|  | 24.75 | 23.53 | 24.31 |
| Rarely/never | 37 | 22 | 59 |
|  | 12.21 | 12.94 | 12.47 |
| Total | 303 | 170 | 473 |
|  | 100.00 | 100.00 | 100.00 |
| Pearson Chi2 = 5.96 Prob = 0.2020 | | | |

First row has *frequencies* and second row has *column percentages*

**Tabulation of Limping Group when TIMEPOINT is 1-year**

| Limping | Group | | |
| --- | --- | --- | --- |
|  | Anterior | Posterior | Total |
| All of the time | 123 | 66 | 189 |
|  | 36.39 | 36.07 | 36.28 |
| Most of the time | 121 | 67 | 188 |
|  | 35.80 | 36.61 | 36.08 |
| Often | 73 | 34 | 107 |
|  | 21.60 | 18.58 | 20.54 |
| Sometimes | 18 | 9 | 27 |
|  | 5.33 | 4.92 | 5.18 |
| Rarely/never | 2 | 6 | 8 |
|  | 0.59 | 3.28 | 1.54 |
| 5 | 1 | 1 | 2 |
|  | 0.30 | 0.55 | 0.38 |
| Total | 338 | 183 | 521 |
|  | 100.00 | 100.00 | 100.00 |
| Pearson Chi2 = 6.37 Prob = 0.2722 | | | |

First row has *frequencies* and second row has *column percentages*

**Tabulation of WalkBeforePain Group when TIMEPOINT is Pre-operatively**

| WalkBeforePain | Group | | |
| --- | --- | --- | --- |
|  | Anterior | Posterior | Total |
| Not at all | 100 | 46 | 146 |
|  | 40.65 | 34.59 | 38.52 |
| Around the house only | 2 | 1 | 3 |
|  | 0.81 | 0.75 | 0.79 |
| 5 to 15 minutes | 6 | 7 | 13 |
|  | 2.44 | 5.26 | 3.43 |
| 16 to 30 minutes | 22 | 21 | 43 |
|  | 8.94 | 15.79 | 11.35 |
| No pain for 30 minutes or more | 116 | 58 | 174 |
|  | 47.15 | 43.61 | 45.91 |
| Total | 246 | 133 | 379 |
|  | 100.00 | 100.00 | 100.00 |
| Pearson Chi2 = 6.64 Prob = 0.1563 | | | |

First row has *frequencies* and second row has *column percentages*

**Tabulation of WalkBeforePain Group when TIMEPOINT is 6-weeks**

| WalkBeforePain | Group | | |
| --- | --- | --- | --- |
|  | Anterior | Posterior | Total |
| Not at all | 70 | 53 | 123 |
|  | 26.02 | 33.97 | 28.94 |
| Around the house only | 9 | 3 | 12 |
|  | 3.35 | 1.92 | 2.82 |
| 5 to 15 minutes | 27 | 10 | 37 |
|  | 10.04 | 6.41 | 8.71 |
| 16 to 30 minutes | 57 | 46 | 103 |
|  | 21.19 | 29.49 | 24.24 |
| No pain for 30 minutes or more | 106 | 44 | 150 |
|  | 39.41 | 28.21 | 35.29 |
| Total | 269 | 156 | 425 |
|  | 100.00 | 100.00 | 100.00 |
| Pearson Chi2 = 10.67 Prob = 0.0305 | | | |

First row has *frequencies* and second row has *column percentages*

**Tabulation of WalkBeforePain Group when TIMEPOINT is 6-months**

| WalkBeforePain | Group | | |
| --- | --- | --- | --- |
|  | Anterior | Posterior | Total |
| Not at all | 57 | 42 | 99 |
|  | 18.81 | 24.71 | 20.93 |
| Around the house only | 14 | 13 | 27 |
|  | 4.62 | 7.65 | 5.71 |
| 5 to 15 minutes | 76 | 35 | 111 |
|  | 25.08 | 20.59 | 23.47 |
| 16 to 30 minutes | 88 | 42 | 130 |
|  | 29.04 | 24.71 | 27.48 |
| No pain for 30 minutes or more | 68 | 38 | 106 |
|  | 22.44 | 22.35 | 22.41 |
| Total | 303 | 170 | 473 |
|  | 100.00 | 100.00 | 100.00 |
| Pearson Chi2 = 5.24 Prob = 0.2637 | | | |

First row has *frequencies* and second row has *column percentages*

**Tabulation of WalkBeforePain Group when TIMEPOINT is 1-year**

| WalkBeforePain | Group | | |
| --- | --- | --- | --- |
|  | Anterior | Posterior | Total |
| Not at all | 124 | 63 | 187 |
|  | 36.69 | 34.43 | 35.89 |
| Around the house only | 80 | 50 | 130 |
|  | 23.67 | 27.32 | 24.95 |
| 5 to 15 minutes | 80 | 40 | 120 |
|  | 23.67 | 21.86 | 23.03 |
| 16 to 30 minutes | 43 | 22 | 65 |
|  | 12.72 | 12.02 | 12.48 |
| No pain for 30 minutes or more | 10 | 7 | 17 |
|  | 2.96 | 3.83 | 3.26 |
| 5 | 1 | 1 | 2 |
|  | 0.30 | 0.55 | 0.38 |
| Total | 338 | 183 | 521 |
|  | 100.00 | 100.00 | 100.00 |
| Pearson Chi2 = 1.49 Prob = 0.9145 | | | |

First row has *frequencies* and second row has *column percentages*

**Tabulation of Stairs Group when TIMEPOINT is Pre-operatively**

| Stairs | Group | | |
| --- | --- | --- | --- |
|  | Anterior | Posterior | Total |
| No, impossible | 98 | 44 | 142 |
|  | 39.84 | 33.08 | 37.47 |
| With extreme difficulty | 6 | 5 | 11 |
|  | 2.44 | 3.76 | 2.90 |
| With moderate difficulty | 11 | 8 | 19 |
|  | 4.47 | 6.02 | 5.01 |
| With little difficulty | 25 | 21 | 46 |
|  | 10.16 | 15.79 | 12.14 |
| Yes, easily | 106 | 55 | 161 |
|  | 43.09 | 41.35 | 42.48 |
| Total | 246 | 133 | 379 |
|  | 100.00 | 100.00 | 100.00 |
| Pearson Chi2 = 4.29 Prob = 0.3678 | | | |

First row has *frequencies* and second row has *column percentages*

**Tabulation of Stairs Group when TIMEPOINT is 6-weeks**

| Stairs | Group | | |
| --- | --- | --- | --- |
|  | Anterior | Posterior | Total |
| No, impossible | 69 | 49 | 118 |
|  | 25.65 | 31.41 | 27.76 |
| With extreme difficulty | 13 | 8 | 21 |
|  | 4.83 | 5.13 | 4.94 |
| With moderate difficulty | 27 | 8 | 35 |
|  | 10.04 | 5.13 | 8.24 |
| With little difficulty | 63 | 52 | 115 |
|  | 23.42 | 33.33 | 27.06 |
| Yes, easily | 97 | 39 | 136 |
|  | 36.06 | 25.00 | 32.00 |
| Total | 269 | 156 | 425 |
|  | 100.00 | 100.00 | 100.00 |
| Pearson Chi2 = 11.45 Prob = 0.0220 | | | |

First row has *frequencies* and second row has *column percentages*

**Tabulation of Stairs Group when TIMEPOINT is 6-months**

| Stairs | Group | | |
| --- | --- | --- | --- |
|  | Anterior | Posterior | Total |
| No, impossible | 34 | 26 | 60 |
|  | 11.22 | 15.29 | 12.68 |
| With extreme difficulty | 22 | 23 | 45 |
|  | 7.26 | 13.53 | 9.51 |
| With moderate difficulty | 70 | 42 | 112 |
|  | 23.10 | 24.71 | 23.68 |
| With little difficulty | 83 | 40 | 123 |
|  | 27.39 | 23.53 | 26.00 |
| Yes, easily | 93 | 39 | 132 |
|  | 30.69 | 22.94 | 27.91 |
| 5 | 1 | 0 | 1 |
|  | 0.33 | 0.00 | 0.21 |
| Total | 303 | 170 | 473 |
|  | 100.00 | 100.00 | 100.00 |
| Pearson Chi2 = 9.57 Prob = 0.0883 | | | |

First row has *frequencies* and second row has *column percentages*

**Tabulation of Stairs Group when TIMEPOINT is 1-year**

| Stairs | Group | | |
| --- | --- | --- | --- |
|  | Anterior | Posterior | Total |
| No, impossible | 108 | 60 | 168 |
|  | 31.95 | 32.79 | 32.25 |
| With extreme difficulty | 100 | 60 | 160 |
|  | 29.59 | 32.79 | 30.71 |
| With moderate difficulty | 81 | 44 | 125 |
|  | 23.96 | 24.04 | 23.99 |
| With little difficulty | 38 | 13 | 51 |
|  | 11.24 | 7.10 | 9.79 |
| Yes, easily | 10 | 5 | 15 |
|  | 2.96 | 2.73 | 2.88 |
| 5 | 1 | 1 | 2 |
|  | 0.30 | 0.55 | 0.38 |
| Total | 338 | 183 | 521 |
|  | 100.00 | 100.00 | 100.00 |
| Pearson Chi2 = 2.71 Prob = 0.7438 | | | |

First row has *frequencies* and second row has *column percentages*

**Tabulation of Socks Group when TIMEPOINT is Pre-operatively**

| Socks | Group | | |
| --- | --- | --- | --- |
|  | Anterior | Posterior | Total |
| No, impossible | 107 | 53 | 160 |
|  | 43.50 | 39.85 | 42.22 |
| With extreme difficulty | 7 | 6 | 13 |
|  | 2.85 | 4.51 | 3.43 |
| With moderate difficulty | 15 | 12 | 27 |
|  | 6.10 | 9.02 | 7.12 |
| With little difficulty | 44 | 23 | 67 |
|  | 17.89 | 17.29 | 17.68 |
| Yes, easily | 73 | 39 | 112 |
|  | 29.67 | 29.32 | 29.55 |
| Total | 246 | 133 | 379 |
|  | 100.00 | 100.00 | 100.00 |
| Pearson Chi2 = 2.03 Prob = 0.7307 | | | |

First row has *frequencies* and second row has *column percentages*

**Tabulation of Socks Group when TIMEPOINT is 6-weeks**

| Socks | Group | | |
| --- | --- | --- | --- |
|  | Anterior | Posterior | Total |
| No, impossible | 83 | 65 | 148 |
|  | 30.86 | 41.67 | 34.82 |
| With extreme difficulty | 12 | 13 | 25 |
|  | 4.46 | 8.33 | 5.88 |
| With moderate difficulty | 39 | 10 | 49 |
|  | 14.50 | 6.41 | 11.53 |
| With little difficulty | 75 | 42 | 117 |
|  | 27.88 | 26.92 | 27.53 |
| Yes, easily | 60 | 26 | 86 |
|  | 22.30 | 16.67 | 20.24 |
| Total | 269 | 156 | 425 |
|  | 100.00 | 100.00 | 100.00 |
| Pearson Chi2 = 13.02 Prob = 0.0112 | | | |

First row has *frequencies* and second row has *column percentages*

**Tabulation of Socks Group when TIMEPOINT is 6-months**

| Socks | Group | | |
| --- | --- | --- | --- |
|  | Anterior | Posterior | Total |
| No, impossible | 87 | 59 | 146 |
|  | 28.71 | 34.71 | 30.87 |
| With extreme difficulty | 22 | 14 | 36 |
|  | 7.26 | 8.24 | 7.61 |
| With moderate difficulty | 64 | 42 | 106 |
|  | 21.12 | 24.71 | 22.41 |
| With little difficulty | 100 | 41 | 141 |
|  | 33.00 | 24.12 | 29.81 |
| Yes, easily | 30 | 14 | 44 |
|  | 9.90 | 8.24 | 9.30 |
| Total | 303 | 170 | 473 |
|  | 100.00 | 100.00 | 100.00 |
| Pearson Chi2 = 5.24 Prob = 0.2639 | | | |

First row has *frequencies* and second row has *column percentages*

**Tabulation of Socks Group when TIMEPOINT is 1-year**

| Socks | Group | | |
| --- | --- | --- | --- |
|  | Anterior | Posterior | Total |
| No, impossible | 126 | 62 | 188 |
|  | 37.28 | 33.88 | 36.08 |
| With extreme difficulty | 134 | 77 | 211 |
|  | 39.64 | 42.08 | 40.50 |
| With moderate difficulty | 57 | 29 | 86 |
|  | 16.86 | 15.85 | 16.51 |
| With little difficulty | 17 | 11 | 28 |
|  | 5.03 | 6.01 | 5.37 |
| Yes, easily | 3 | 3 | 6 |
|  | 0.89 | 1.64 | 1.15 |
| 5 | 1 | 1 | 2 |
|  | 0.30 | 0.55 | 0.38 |
| Total | 338 | 183 | 521 |
|  | 100.00 | 100.00 | 100.00 |
| Pearson Chi2 = 1.62 Prob = 0.8992 | | | |

First row has *frequencies* and second row has *column percentages*

**Tabulation of StandAfterMeal Group when TIMEPOINT is Pre-operatively**

| StandAfterMeal | Group | | |
| --- | --- | --- | --- |
|  | Anterior | Posterior | Total |
| Unbearable | 96 | 43 | 139 |
|  | 39.02 | 32.33 | 36.68 |
| Very painful | 1 | 2 | 3 |
|  | 0.41 | 1.50 | 0.79 |
| Moderately painful | 13 | 7 | 20 |
|  | 5.28 | 5.26 | 5.28 |
| Slightly painful | 34 | 27 | 61 |
|  | 13.82 | 20.30 | 16.09 |
| Not at all painful | 102 | 54 | 156 |
|  | 41.46 | 40.60 | 41.16 |
| Total | 246 | 133 | 379 |
|  | 100.00 | 100.00 | 100.00 |
| Pearson Chi2 = 4.64 Prob = 0.3268 | | | |

First row has *frequencies* and second row has *column percentages*

**Tabulation of StandAfterMeal Group when TIMEPOINT is 6-weeks**

| StandAfterMeal | Group | | |
| --- | --- | --- | --- |
|  | Anterior | Posterior | Total |
| Unbearable | 62 | 48 | 110 |
|  | 23.05 | 30.77 | 25.88 |
| Very painful | 9 | 4 | 13 |
|  | 3.35 | 2.56 | 3.06 |
| Moderately painful | 25 | 10 | 35 |
|  | 9.29 | 6.41 | 8.24 |
| Slightly painful | 76 | 49 | 125 |
|  | 28.25 | 31.41 | 29.41 |
| Not at all painful | 97 | 45 | 142 |
|  | 36.06 | 28.85 | 33.41 |
| Total | 269 | 156 | 425 |
|  | 100.00 | 100.00 | 100.00 |
| Pearson Chi2 = 5.34 Prob = 0.2541 | | | |

First row has *frequencies* and second row has *column percentages*

**Tabulation of StandAfterMeal Group when TIMEPOINT is 6-months**

| StandAfterMeal | Group | | |
| --- | --- | --- | --- |
|  | Anterior | Posterior | Total |
| Unbearable | 27 | 14 | 41 |
|  | 8.91 | 8.24 | 8.67 |
| Very painful | 7 | 9 | 16 |
|  | 2.31 | 5.29 | 3.38 |
| Moderately painful | 64 | 49 | 113 |
|  | 21.12 | 28.82 | 23.89 |
| Slightly painful | 119 | 52 | 171 |
|  | 39.27 | 30.59 | 36.15 |
| Not at all painful | 86 | 46 | 132 |
|  | 28.38 | 27.06 | 27.91 |
| Total | 303 | 170 | 473 |
|  | 100.00 | 100.00 | 100.00 |
| Pearson Chi2 = 7.97 Prob = 0.0927 | | | |

First row has *frequencies* and second row has *column percentages*

**Tabulation of StandAfterMeal Group when TIMEPOINT is 1-year**

| StandAfterMeal | Group | | |
| --- | --- | --- | --- |
|  | Anterior | Posterior | Total |
| Unbearable | 80 | 34 | 114 |
|  | 23.67 | 18.58 | 21.88 |
| Very painful | 148 | 97 | 245 |
|  | 43.79 | 53.01 | 47.02 |
| Moderately painful | 80 | 37 | 117 |
|  | 23.67 | 20.22 | 22.46 |
| Slightly painful | 28 | 11 | 39 |
|  | 8.28 | 6.01 | 7.49 |
| Not at all painful | 2 | 3 | 5 |
|  | 0.59 | 1.64 | 0.96 |
| 5 | 0 | 1 | 1 |
|  | 0.00 | 0.55 | 0.19 |
| Total | 338 | 183 | 521 |
|  | 100.00 | 100.00 | 100.00 |
| Pearson Chi2 = 8.20 Prob = 0.1453 | | | |

First row has *frequencies* and second row has *column percentages*

**Tabulation of Car Group when TIMEPOINT is Pre-operatively**

| Car | Group | | |
| --- | --- | --- | --- |
|  | Anterior | Posterior | Total |
| Impossible to do | 98 | 45 | 143 |
|  | 39.84 | 33.83 | 37.73 |
| Extreme difficulty | 4 | 4 | 8 |
|  | 1.63 | 3.01 | 2.11 |
| Moderate trouble | 13 | 14 | 27 |
|  | 5.28 | 10.53 | 7.12 |
| Very little trouble | 27 | 19 | 46 |
|  | 10.98 | 14.29 | 12.14 |
| No trouble at all | 104 | 51 | 155 |
|  | 42.28 | 38.35 | 40.90 |
| Total | 246 | 133 | 379 |
|  | 100.00 | 100.00 | 100.00 |
| Pearson Chi2 = 6.04 Prob = 0.1962 | | | |

First row has *frequencies* and second row has *column percentages*

**Tabulation of Car Group when TIMEPOINT is 6-weeks**

| Car | Group | | |
| --- | --- | --- | --- |
|  | Anterior | Posterior | Total |
| Impossible to do | 68 | 52 | 120 |
|  | 25.28 | 33.33 | 28.24 |
| Extreme difficulty | 10 | 6 | 16 |
|  | 3.72 | 3.85 | 3.76 |
| Moderate trouble | 19 | 15 | 34 |
|  | 7.06 | 9.62 | 8.00 |
| Very little trouble | 65 | 36 | 101 |
|  | 24.16 | 23.08 | 23.76 |
| No trouble at all | 107 | 47 | 154 |
|  | 39.78 | 30.13 | 36.24 |
| Total | 269 | 156 | 425 |
|  | 100.00 | 100.00 | 100.00 |
| Pearson Chi2 = 5.66 Prob = 0.2258 | | | |

First row has *frequencies* and second row has *column percentages*

**Tabulation of Car Group when TIMEPOINT is 6-months**

| Car | Group | | |
| --- | --- | --- | --- |
|  | Anterior | Posterior | Total |
| Impossible to do | 47 | 32 | 79 |
|  | 15.51 | 18.82 | 16.70 |
| Extreme difficulty | 39 | 21 | 60 |
|  | 12.87 | 12.35 | 12.68 |
| Moderate trouble | 43 | 28 | 71 |
|  | 14.19 | 16.47 | 15.01 |
| Very little trouble | 110 | 65 | 175 |
|  | 36.30 | 38.24 | 37.00 |
| No trouble at all | 64 | 24 | 88 |
|  | 21.12 | 14.12 | 18.60 |
| Total | 303 | 170 | 473 |
|  | 100.00 | 100.00 | 100.00 |
| Pearson Chi2 = 4.10 Prob = 0.3931 | | | |

First row has *frequencies* and second row has *column percentages*

**Tabulation of Car Group when TIMEPOINT is 1-year**

| Car | Group | | |
| --- | --- | --- | --- |
|  | Anterior | Posterior | Total |
| Impossible to do | 209 | 112 | 321 |
|  | 61.83 | 61.20 | 61.61 |
| Extreme difficulty | 86 | 48 | 134 |
|  | 25.44 | 26.23 | 25.72 |
| Moderate trouble | 30 | 17 | 47 |
|  | 8.88 | 9.29 | 9.02 |
| Very little trouble | 12 | 5 | 17 |
|  | 3.55 | 2.73 | 3.26 |
| No trouble at all | 1 | 0 | 1 |
|  | 0.30 | 0.00 | 0.19 |
| 5 | 0 | 1 | 1 |
|  | 0.00 | 0.55 | 0.19 |
| Total | 338 | 183 | 521 |
|  | 100.00 | 100.00 | 100.00 |
| Pearson Chi2 = 2.69 Prob = 0.7475 | | | |

First row has *frequencies* and second row has *column percentages*

**Tabulation of Washing Group when TIMEPOINT is Pre-operatively**

| Washing | Group | | |
| --- | --- | --- | --- |
|  | Anterior | Posterior | Total |
| Impossible to do | 96 | 42 | 138 |
|  | 39.02 | 31.58 | 36.41 |
| Extreme difficulty | 3 | 3 | 6 |
|  | 1.22 | 2.26 | 1.58 |
| Moderate trouble | 13 | 12 | 25 |
|  | 5.28 | 9.02 | 6.60 |
| Very little trouble | 17 | 17 | 34 |
|  | 6.91 | 12.78 | 8.97 |
| No trouble at all | 117 | 59 | 176 |
|  | 47.56 | 44.36 | 46.44 |
| Total | 246 | 133 | 379 |
|  | 100.00 | 100.00 | 100.00 |
| Pearson Chi2 = 7.24 Prob = 0.1239 | | | |

First row has *frequencies* and second row has *column percentages*

**Tabulation of Washing Group when TIMEPOINT is 6-weeks**

| Washing | Group | | |
| --- | --- | --- | --- |
|  | Anterior | Posterior | Total |
| Impossible to do | 66 | 48 | 114 |
|  | 24.54 | 30.77 | 26.82 |
| Extreme difficulty | 6 | 2 | 8 |
|  | 2.23 | 1.28 | 1.88 |
| Moderate trouble | 24 | 20 | 44 |
|  | 8.92 | 12.82 | 10.35 |
| Very little trouble | 44 | 30 | 74 |
|  | 16.36 | 19.23 | 17.41 |
| No trouble at all | 129 | 56 | 185 |
|  | 47.96 | 35.90 | 43.53 |
| Total | 269 | 156 | 425 |
|  | 100.00 | 100.00 | 100.00 |
| Pearson Chi2 = 7.12 Prob = 0.1298 | | | |

First row has *frequencies* and second row has *column percentages*

**Tabulation of Washing Group when TIMEPOINT is 6-months**

| Washing | Group | | |
| --- | --- | --- | --- |
|  | Anterior | Posterior | Total |
| Impossible to do | 40 | 12 | 52 |
|  | 13.20 | 7.06 | 10.99 |
| Extreme difficulty | 11 | 13 | 24 |
|  | 3.63 | 7.65 | 5.07 |
| Moderate trouble | 65 | 40 | 105 |
|  | 21.45 | 23.53 | 22.20 |
| Very little trouble | 58 | 35 | 93 |
|  | 19.14 | 20.59 | 19.66 |
| No trouble at all | 129 | 70 | 199 |
|  | 42.57 | 41.18 | 42.07 |
| Total | 303 | 170 | 473 |
|  | 100.00 | 100.00 | 100.00 |
| Pearson Chi2 = 7.58 Prob = 0.1083 | | | |

First row has *frequencies* and second row has *column percentages*

**Tabulation of Washing Group when TIMEPOINT is 1-year**

| Washing | Group | | |
| --- | --- | --- | --- |
|  | Anterior | Posterior | Total |
| Impossible to do | 138 | 70 | 208 |
|  | 40.83 | 38.25 | 39.92 |
| Extreme difficulty | 89 | 55 | 144 |
|  | 26.33 | 30.05 | 27.64 |
| Moderate trouble | 76 | 48 | 124 |
|  | 22.49 | 26.23 | 23.80 |
| Very little trouble | 12 | 5 | 17 |
|  | 3.55 | 2.73 | 3.26 |
| No trouble at all | 22 | 4 | 26 |
|  | 6.51 | 2.19 | 4.99 |
| 5 | 1 | 1 | 2 |
|  | 0.30 | 0.55 | 0.38 |
| Total | 338 | 183 | 521 |
|  | 100.00 | 100.00 | 100.00 |
| Pearson Chi2 = 6.38 Prob = 0.2713 | | | |

First row has *frequencies* and second row has *column percentages*

**Tabulation of Shopping Group when TIMEPOINT is Pre-operatively**

| Shopping | Group | | |
| --- | --- | --- | --- |
|  | Anterior | Posterior | Total |
| No, impossible | 97 | 44 | 141 |
|  | 39.43 | 33.08 | 37.20 |
| With extreme difficulty | 3 | 2 | 5 |
|  | 1.22 | 1.50 | 1.32 |
| With moderate difficulty | 13 | 12 | 25 |
|  | 5.28 | 9.02 | 6.60 |
| With little difficulty | 18 | 22 | 40 |
|  | 7.32 | 16.54 | 10.55 |
| Yes, easily | 115 | 53 | 168 |
|  | 46.75 | 39.85 | 44.33 |
| Total | 246 | 133 | 379 |
|  | 100.00 | 100.00 | 100.00 |
| Pearson Chi2 = 10.70 Prob = 0.0301 | | | |

First row has *frequencies* and second row has *column percentages*

**Tabulation of Shopping Group when TIMEPOINT is 6-weeks**

| Shopping | Group | | |
| --- | --- | --- | --- |
|  | Anterior | Posterior | Total |
| No, impossible | 69 | 52 | 121 |
|  | 25.65 | 33.33 | 28.47 |
| With extreme difficulty | 8 | 3 | 11 |
|  | 2.97 | 1.92 | 2.59 |
| With moderate difficulty | 25 | 15 | 40 |
|  | 9.29 | 9.62 | 9.41 |
| With little difficulty | 60 | 41 | 101 |
|  | 22.30 | 26.28 | 23.76 |
| Yes, easily | 107 | 45 | 152 |
|  | 39.78 | 28.85 | 35.76 |
| Total | 269 | 156 | 425 |
|  | 100.00 | 100.00 | 100.00 |
| Pearson Chi2 = 6.44 Prob = 0.1689 | | | |

First row has *frequencies* and second row has *column percentages*

**Tabulation of Shopping Group when TIMEPOINT is 6-months**

| Shopping | Group | | |
| --- | --- | --- | --- |
|  | Anterior | Posterior | Total |
| No, impossible | 38 | 22 | 60 |
|  | 12.54 | 12.94 | 12.68 |
| With extreme difficulty | 29 | 20 | 49 |
|  | 9.57 | 11.76 | 10.36 |
| With moderate difficulty | 68 | 51 | 119 |
|  | 22.44 | 30.00 | 25.16 |
| With little difficulty | 105 | 45 | 150 |
|  | 34.65 | 26.47 | 31.71 |
| Yes, easily | 63 | 32 | 95 |
|  | 20.79 | 18.82 | 20.08 |
| Total | 303 | 170 | 473 |
|  | 100.00 | 100.00 | 100.00 |
| Pearson Chi2 = 5.50 Prob = 0.2396 | | | |

First row has *frequencies* and second row has *column percentages*

**Tabulation of Shopping Group when TIMEPOINT is 1-year**

| Shopping | Group | | |
| --- | --- | --- | --- |
|  | Anterior | Posterior | Total |
| No, impossible | 120 | 76 | 196 |
|  | 35.50 | 41.53 | 37.62 |
| With extreme difficulty | 145 | 70 | 215 |
|  | 42.90 | 38.25 | 41.27 |
| With moderate difficulty | 59 | 28 | 87 |
|  | 17.46 | 15.30 | 16.70 |
| With little difficulty | 13 | 8 | 21 |
|  | 3.85 | 4.37 | 4.03 |
| 5 | 1 | 1 | 2 |
|  | 0.30 | 0.55 | 0.38 |
| Total | 338 | 183 | 521 |
|  | 100.00 | 100.00 | 100.00 |
| Pearson Chi2 = 2.37 Prob = 0.6674 | | | |

First row has *frequencies* and second row has *column percentages*

**Tabulation of PainInterferWork Group when TIMEPOINT is Pre-operatively**

| PainInterferWork | Group | | |
| --- | --- | --- | --- |
|  | Anterior | Posterior | Total |
| No, impossible | 110 | 50 | 160 |
|  | 44.72 | 37.59 | 42.22 |
| With extreme difficulty | 8 | 9 | 17 |
|  | 3.25 | 6.77 | 4.49 |
| With moderate difficulty | 17 | 12 | 29 |
|  | 6.91 | 9.02 | 7.65 |
| With little difficulty | 17 | 15 | 32 |
|  | 6.91 | 11.28 | 8.44 |
| Yes, easily | 94 | 47 | 141 |
|  | 38.21 | 35.34 | 37.20 |
| Total | 246 | 133 | 379 |
|  | 100.00 | 100.00 | 100.00 |
| Pearson Chi2 = 6.06 Prob = 0.1947 | | | |

First row has *frequencies* and second row has *column percentages*

**Tabulation of PainInterferWork Group when TIMEPOINT is 6-weeks**

| PainInterferWork | Group | | |
| --- | --- | --- | --- |
|  | Anterior | Posterior | Total |
| No, impossible | 74 | 55 | 129 |
|  | 27.51 | 35.26 | 30.35 |
| With extreme difficulty | 14 | 12 | 26 |
|  | 5.20 | 7.69 | 6.12 |
| With moderate difficulty | 30 | 18 | 48 |
|  | 11.15 | 11.54 | 11.29 |
| With little difficulty | 46 | 27 | 73 |
|  | 17.10 | 17.31 | 17.18 |
| Yes, easily | 105 | 44 | 149 |
|  | 39.03 | 28.21 | 35.06 |
| Total | 269 | 156 | 425 |
|  | 100.00 | 100.00 | 100.00 |
| Pearson Chi2 = 6.27 Prob = 0.1799 | | | |

First row has *frequencies* and second row has *column percentages*

**Tabulation of PainInterferWork Group when TIMEPOINT is 6-months**

| PainInterferWork | Group | | |
| --- | --- | --- | --- |
|  | Anterior | Posterior | Total |
| No, impossible | 53 | 25 | 78 |
|  | 17.49 | 14.71 | 16.49 |
| With extreme difficulty | 35 | 23 | 58 |
|  | 11.55 | 13.53 | 12.26 |
| With moderate difficulty | 85 | 48 | 133 |
|  | 28.05 | 28.24 | 28.12 |
| With little difficulty | 63 | 35 | 98 |
|  | 20.79 | 20.59 | 20.72 |
| Yes, easily | 67 | 39 | 106 |
|  | 22.11 | 22.94 | 22.41 |
| Total | 303 | 170 | 473 |
|  | 100.00 | 100.00 | 100.00 |
| Pearson Chi2 = 0.90 Prob = 0.9250 | | | |

First row has *frequencies* and second row has *column percentages*

**Tabulation of PainInterferWork Group when TIMEPOINT is 1-year**

| PainInterferWork | Group | | |
| --- | --- | --- | --- |
|  | Anterior | Posterior | Total |
| No, impossible | 190 | 88 | 278 |
|  | 56.21 | 48.09 | 53.36 |
| With extreme difficulty | 93 | 59 | 152 |
|  | 27.51 | 32.24 | 29.17 |
| With moderate difficulty | 50 | 30 | 80 |
|  | 14.79 | 16.39 | 15.36 |
| With little difficulty | 1 | 3 | 4 |
|  | 0.30 | 1.64 | 0.77 |
| Yes, easily | 3 | 2 | 5 |
|  | 0.89 | 1.09 | 0.96 |
| 5 | 1 | 1 | 2 |
|  | 0.30 | 0.55 | 0.38 |
| Total | 338 | 183 | 521 |
|  | 100.00 | 100.00 | 100.00 |
| Pearson Chi2 = 5.61 Prob = 0.3457 | | | |

First row has *frequencies* and second row has *column percentages*
